# Breast cancer worry, uncertainty, and perceived risk following breast density notification in a longitudinal mammography screening cohort

**DOI:** 10.1186/s13058-022-01584-2

**Published:** 2022-12-21

**Authors:** Erica J. Lee Argov, Carmen B. Rodriguez, Mariangela Agovino, Ying Wei, Rachel C. Shelton, Rita Kukafka, Karen M. Schmitt, Elise Desperito, Mary Beth Terry, Parisa Tehranifar

**Affiliations:** 1 Department of Epidemiology, Columbia University Mailman School of Public Health, 722 W 168th St., New York, NY USA; 2 Department of Biostatistics, Columbia University Mailman School of Public Health, 722 W 168th St., New York, NY USA; 3 Department of Sociomedical Sciences, Columbia University Mailman School of Public Health, 722 W 168th St., New York, NY USA; 4 Herbert Irving Comprehensive Cancer Center, Columbia University Irving Medical Center, 161 Fort Washington Ave., New York, NY USA; 5 Department of Biomedical Informatics, Vagelos College of Physicians and Surgeons, Columbia University, 622 West 168th St., New York, NY USA; 6 Division of Academics, Columbia University School of Nursing, New York, NY USA; 7 Department of Radiology, Columbia University Irving Medical Center, New York, NY USA

**Keywords:** Breast density notification, Breast density awareness, Perceived risk, Breast cancer uncertainty

## Abstract

**Background:**

Dense breast notification (DBN) legislation aims to increase a woman’s awareness of her personal breast density and the implications of having dense breasts for breast cancer detection and risk. This information may adversely affect women’s breast cancer worry, perceptions of risk, and uncertainty about screening, which may persist over time or vary by sociodemographic factors. We examined short- and long-term psychological responses to DBN and awareness of breast density (BD).

**Methods:**

In a predominantly Hispanic New York City screening cohort (63% Spanish-speaking), ages 40–60 years, we assessed breast cancer worry, perceived breast cancer risk, and uncertainties about breast cancer risk and screening choices, in short (1–3 months)- and long-term (9–18 months) surveys following the enrollment screening mammogram (between 2016 and 2018). We compared psychological responses by women’s dense breast status (as a proxy for DBN receipt) and BD awareness and examined multiplicative interaction by education, health literacy, nativity, and preferred interview language.

**Results:**

In multivariable models using short-term surveys, BD awareness was associated with increased perceived risk (odds ratio (OR) 2.27, 95% confidence interval (CI) 0.99, 5.20 for high, OR 2.19, 95% CI 1.34, 3.58 for moderate, vs. low risk) in the overall sample, and with increased uncertainty about risk (OR 1.97 per 1-unit increase, 95% CI 1.15, 3.39) and uncertainty about screening choices (OR 1.73 per 1-unit increase, 95% CI 1.01, 2.9) in Spanish-speaking women. DBN was associated with decreased perceived risk among women with at least some college education (OR 0.32, 95% CI 0.11, 0.89, for high, OR 0.50, 95% CI 0.29, 0.89, for moderate vs. low risk), while those with a high school education or less experienced an increase (OR 3.01, 95% CI 1.05, 8.67 high vs. low risk). There were no associations observed between DBN or BD awareness and short-term breast cancer worry, nor with any psychological outcomes at long-term surveys.

**Conclusions:**

Associations of BD awareness and notification with breast cancer-related psychological outcomes were limited to short-term increases in perceived breast cancer risk dependent on educational attainment, and increases in uncertainty around breast cancer risk and screening choices among Spanish-speaking women.

**Supplementary Information:**

The online version contains supplementary material available at 10.1186/s13058-022-01584-2.

## Background

High mammographic breast density (BD)—relatively large opaque white areas of mostly fibroglandular tissue on a mammogram—not only raises breast cancer risk but also makes it more difficult for radiologists to detect breast cancer on a mammogram [1, 2]. These factors prompted the passage of state-level legislation designed to inform women if dense breasts were identified on their screening mammogram and a federal amendment to the Mammography Quality Standards Act to mandate the disclosure of BD information to women and their providers [3, 4]. The goal of most dense breast notification (DBN) legislation is to increase awareness of women’s own BD and BD’s impact on breast cancer detection and risk and to inform women of their potential need to obtain supplemental imaging screening via ultrasound and/or magnetic resonance imaging (MRI) techniques, for example [5–7].

In addition to structural factors such as healthcare access and coverage of costs for supplemental testing, any effect of DBN on breast cancer screening behavior is contingent upon increasing women’s awareness of breast density and its implications as well as women’s cognitive and emotional appraisal of this information [6]. A recent systematic review emphasized that overall, BD awareness tends to be low, and some, but not all, studies found that women residing in states with DBN laws report greater knowledge of general or personal breast density, slightly increased knowledge of density’s masking effect, and no changes in knowledge of the increased risk of high BD on breast cancer [6, 7]. Fewer studies have examined psychological appraisal and responses to DBN. The lack of consensus on screening recommendations for women with dense breasts and providers’ limited training in breast density counseling [8, 9] have raised concerns that DBN may trigger negative emotional and cognitive responses, such as increasing inaccurate risk perceptions, worry, and uncertainty about breast cancer screening choices that may negatively impact women and in turn inadvertently reduce screening participation [7, 10, 11]. Qualitative studies have found some level of increased anxiety or worry as well as confusion over the next steps, while cross-sectional surveys and one study of pre-/post-legislation data revealed no difference in breast cancer worry or fear by DBN [6, 7]. Research is also lacking as to whether any initial psychological reactions in response to notification persist long enough to influence screening behavior at the time of the next screening mammogram [12]. Finally, although prior research has found that Hispanic/Latina (hereafter Hispanic) women, women with lower education levels and with limited English proficiency have lower understanding of breast density, limited empirical research has examined whether women’s psychological responses to DBN may also vary by these factors [12–15]. Investigating these questions in racially, ethnically, and educationally diverse populations can provide important data for identifying population groups that may benefit from additional educational and clinical support.

In a longitudinal study within a majority Hispanic immigrant screening cohort [13], we specifically evaluated whether psychological outcomes such as breast cancer worry, perceived absolute and comparative risk, and uncertainty about breast cancer risk and screening choices varied in short-term (approximately 1–3 months) and long-term (approximately 9–18 months) surveys collected after receiving a screening mammogram according to women’s prior BD awareness and dense breast status as a proxy for DBN. We assumed that women with mammographically dense breasts had received DBN after their baseline screening mammogram and explored modification by individual-level sociodemographic characteristics.

## Methods

The New York Mammographic Density (NY MaDe) Study is a study of breast cancer risk factors and screening in a mammography cohort of women in Northern Manhattan, New York City, and has been previously described [13, 16, 17]. Between 2016 and 2018, we recruited 812 women, ages 40–60 years, during their screening mammography appointment at a clinic in Northern Manhattan. Trained bilingual staff conducted in-person baseline surveys with participants in their preferred language (33% in English, 67% in Spanish) to collect information on sociodemographic characteristics, breast cancer risk factors, breast cancer screening history, and medical and lifestyle history. The questionnaire included questions on women’s awareness and knowledge of breast density and breast cancer-related psychological factors such as worry, uncertainty, and absolute and comparative risk.

We extracted clinical mammography reports since January 2013 to obtain the dense breast status of their screening mammograms post-implementation of New York’s DBN (in 2013). In accordance with New York State legislation, the recruiting mammography clinic provided women with dense breasts the state-mandated additional DBN text along with their clinical mammography results, provided in both English and Spanish [13]. We contacted all participants via mail about 1–3 months (short-term follow-up, mean 1.6 months) from the baseline interview to allow them enough time to receive their baseline mammography reports, and again at 9–18 months (long-term follow-up, mean 14 months) from baseline. Bilingual staff administered these interviews by phone or in-person at the mammography clinic, or surveys were self-administered by mail or electronically through a secure link. The baseline and follow-up surveys included questions about breast cancer-related psychological factors. The short-term follow-up was completed by 612 women (75%), and the long-term follow-up was completed by 630 women (77.6%); 538 women (66.3%) completed both follow-ups, while 166 women (20%) completed the baseline and only one of the follow-up surveys. We excluded 4 women with a history of breast cancer and 1 woman for whom mammography reports could not be obtained. All women who completed any follow-up survey were retained for analysis of that survey (*n* = 607 for short term and 626 for long term).

The Columbia University Medical Center institutional review board approved this study, and all women provided written informed consent prior to data collection.

### Measures

For breast density status, as described previously [13], we retrieved the Breast Imaging Reporting and Data System (BI-RADS) breast composition classification categories for all mammograms since 2013 through electronic medical records. Following the definition used in the breast density notification letter, we categorized each mammogram report as having clinically assessed BI-RADS categories of dense (heterogeneously or extremely dense; categories C or D) or non-dense (almost entirely fatty or scattered fibroglandular; categories A or B) breasts. Our primary measure of DBN used dense breast status as a proxy, based on the dense breast categories for the baseline mammogram report (i.e., mammogram obtained at enrollment and baseline survey). We also assessed all mammogram reports since DBN implementation in New York State in 2013 to capture the number of times women may have received notification. Consistent with prior research [18–20], we examined BD awareness by asking respondents the following at baseline and short term: “Do you know about something called breast density or dense breasts?”, “Have you ever been told that you have dense breasts?” Participants who responded affirmatively to either of these questions were classified as “aware of BD,” and all others were “not aware.” We included responses to this question at short-term to include women who may have been made aware of BD after receiving their baseline mammogram results. The majority of women (83.5%) who were aware of BD at short term were also aware at baseline. In addition to assessing DBN and BD awareness individually, we created a four-level variable to examine the combination of these two variables as follows: aware and non-dense, unaware and non-dense, aware and dense, and unaware and dense. Assessment of participant’s emotional and cognitive appraisal of future breast cancer risk is detailed in Table 1. These questions draw on commonly used and validated measures previously used in this study population [21] including the Lerman Breast Cancer Worry Scale [22], well-studied questions for perceptions of absolute and comparative breast cancer risk [23], and uncertainty about breast cancer risk and screening choices adapted from the uncertainty subscale of the Multidimensional Impact of Cancer Risk Assessment questionnaire [24]. We asked these questions at baseline, short-term, and long-term follow-ups.

**Table 1:** Details of psychological constructs and survey questions

Construct^a^	Survey prompt	Response options	Categorized^b^
Worry [22]	In the past 4 weeks, how often have you worried about getting any of the following illnesses? *Breast cancer*	Rarely or never	Low
		Sometimes	High
		Often	
		All the time	
Perceived absolute risk [23]	How likely do you think it is that you will develop any of the following illnesses in the future? *Breast cancer*	Very low	Low risk
		Somewhat low	Moderate risk
		Moderate	
		Somewhat high	High risk
		Very high	
Perceived comparative risk [23]	Compared to an average woman your age, would you say that you are:	More likely to get breast cancer	
		As likely to get breast cancer	
		Less likely to get breast cancer	
Uncertainty about risk [24]	In the past 4 weeks, how often have you experienced the following because of thoughts and feelings about breast cancer? *I have felt uncertain about my risk for breast cancer*	Never	Ordinally^c^
		Rarely	
		Sometimes	
		Often	
Uncertainty about choices [24]	In the past 4 weeks, how often have you experienced the following because of thoughts and feelings about breast cancer? *I have felt uncertain about what my choices are for screening or early detection of breast cancer*	Never	Ordinally^c^
		Rarely	
		Sometimes	
		Often	

^a^References corresponding to sources for each survey question are indicated next to each construct name

^b^Response options were collapsed due to small numbers in the extreme categories; perceived comparative risk was treated as-is

^c^Proportional odds assumption held for both uncertainty measures; thus, they were treated ordinally

We considered the following covariates obtained from the baseline interview based on prior predictors of BD awareness in this cohort [13]: 5-year absolute Gail risk score [25] calculated using breast cancer risk factors collected during the baseline questionnaire, mammography callback history (called back at least once for additional testing after a screening mammogram vs. never called back), nativity status (US-born vs. foreign born), dominant language (baseline survey language, Spanish or English), health literacy [26], educational attainment (less than high school graduate vs. high school graduate vs. some college or trade school vs. bachelor’s degree or higher), and history of breast biopsy (yes vs. no).

### Statistical analysis

For descriptive analyses, we compared participant characteristics across survey waves using chi-squared or t tests. For multivariable analyses, we first determined the most appropriate models for the outcome data, which contained multiple answer choices. We used binary logistic regression to assess high versus low for breast cancer worry due to small cell sizes when disaggregated. We then tested the validity of the proportional odds assumption for all other psychological outcomes to see if it was appropriate to treat these outcomes as ordinal variables. This assumption held only for the uncertainty measures, so cumulative logistic regression was used. We then used multinomial logistic regression for perceived absolute and comparative risk. We modeled each psychological outcome separately for each wave using the appropriate models described above. To isolate the impact of DBN of baseline mammogram from women’s baseline psychological characteristics, we adjusted for the corresponding psychological factor reported at baseline (e.g., worry was modeled with DBN and awareness at short-term follow-up, adjusted for worry reported at baseline) (base model). To this, Model 1 added separate sets of covariates specific to each outcome variable (Table 3). These covariates were selected if significantly associated with that outcome and with either exposure (Table 2, significant covariates indicated by footnotes). To isolate the effect of DBN from general breast density awareness, we added adjustment for the mutual exposure to Model 1 (i.e., DBN was added to models examining awareness and vice versa) (Model 2). Models for each psychological outcome excluded only those missing responses for that outcome; for example, the perceived risk questions had 6–9% missing between short- and long-term survey waves.

**Table 2:** Characteristics of population responding to short-term follow-up (*n* = 607), by dense breast notification (DBN) and breast density (BD) awareness

Population responding to short-term follow-up	Total *N* = 607	Reported no BD awareness (*n* = 401)	Reported BD awareness (*n* = 206)	No DBN (*n* = 394)	With DBN (*n* = 213)
	*n* (%) or mean (SD)	*n* (%) or mean (SD)	*n* (%) or mean (SD)	*n* (%) or mean (SD)	*n* (%) or mean (SD)
*Age at baseline interview (years)*^*¥α*^	51.6 (5.7)	52.0 (5.5)	50.7 (5.8)	52.3 (5.7)	50.2 (5.4)
*Primary language*^*¥α*^					
English	227 (37.4)	89 (22.2)	138 (67.0)	134 (34.0)	93 (43.7)
Spanish	380 (62.6)	312 (77.8)	68 (33.0)	260 (66.0)	120 (56.3)
*Nativity Status*^*α*^					
US-born	164 (27.0)	59 (14.7)	105 (51.0)	101 (25.6)	63 (29.6)
Foreign Born	443 (73.0)	342 (85.3)	101 (49.0)	293 (74.4)	150 (70.4)
*Race/Ethnicity*^*¥α*^					
Non-Hispanic White	56 (9.2)	9 (2.2)	47 (22.8)	25 (6.4)	31 (14.6)
Non-Hispanic Black	75 (12.4)	30 (7.5)	45 (21.8)	52 (13.2)	23 (10.8)
Non-Hispanic Mixed/ Other	17 (2.8)	8 (2.0)	9 (4.4)	7 (1.8)	10 (4.7)
Hispanic White	151 (24.9)	110 (27.4)	41 (19.9)	103 (26.1)	48 (22.5)
Hispanic Black	120 (19.8)	93 (23.2)	27 (13.1)	82 (20.8)	38 (17.8)
Hispanic Mixed/Other	188 (30.9)	151 (37.7)	37 (18.0)	125 (31.7)	63 (29.6)
*Educational attainment*^*¥α*^					
Bachelor’s degree or higher	201 (33.1)	86 (21.5)	115 (55.8)	107 (27.2)	94 (44.1)
Some College or Trade School	149 (24.6)	98 (24.4)	51 (24.8)	105 (26.7)	44 (20.7)
High School Graduate or less	257 (42.3)	217 (54.1)	40 (19.4)	182 (46.2)	75 (35.2)
*Health Literacy*^*¥*α^	5.4 (3.2)	6.0 (3.4)	4.3 (2.5)	5.7 (3.3)	5.0 (3.0)
Missing	1 (0.2)		1 (0.5)		1 (0.5)
*Breast Biopsy History*^*¥*^	120 (19.8)	74 (18.5)	46 (22.3)	64 (16.2)	56 (26.3)
*1st degree family history of BC*	73 (12.0)	43 (10.7)	30 (14.6)	48 (12.2)	25 (11.7)
Missing	2 (0.3)	1 (0.3)	1 (0.5)	1 (0.3)	1 (0.5)
*Gail 5-year absolute Risk Score*^*α*^	1.0 (0.62)	0.9 (0.6)	1.2 (0.7)	1.0 (0.6)	1.0 (0.6)
*Gail 5-year absolute Risk Score*^*α*^ ≥ 1.67 (high risk)	75 (12.4)	40 (10.0)	35 (17.0)	50 (12.7)	25 (11.7)
*Mammography Callback*^*¥α*^					
*No, never*	326 (53.7)	230 (57.4)	96 (46.6)	230 (58.4)	96 (45.1)
*At least once*	281 (46.3)	171 (42.6)	110 (53.4)	164 (41.6)	117 (54.9)
*Number of dense breast mammograms since legislation year (2013) prior to baseline*^*¥α*^					
0	261 (43.0)	200 (49.9)	61 (29.6)	251 (63.7)	10 (4.7)
1	104 (17.1)	65 (16.2)	39 (18.9)	52 (13.2)	52 (24.4)
≥ 2	134 (22.1)	68 (17.0)	66 (32.0)	27 (6.9)	107 (50.2)
Missing	108 (17.8)	68 (17.0)	40 (19.4)	64 (16.2)	44 (20.7)
*Breast cancer worry at baseline *^*α*^					
Rarely or never	430 (70.8)	299 (74.6)	131 (63.6)	279 (70.8)	151 (70.9)
Sometimes	112 (18.5)	60 (15.0)	52 (25.2)	67 (17.0)	45 (21.1)
Often	62 (10.2)	41 (10.2)	21 (10.2)	45 (11.4)	17 (8.0)
Missing	3 (0.5)	1 (0.3)	2 (1.0)	3 (0.8)	0 (0)
*Perceived absolute risk at baseline*					
Low	300 (49.4)	211 (52.6)	89 (43.2)	201 (51.0)	99 (46.5)
Moderate	252 (41.5)	159 (39.7)	93 (45.2)	161 (40.9)	91 (42.7)
High	52 (8.6)	30 (7.5)	22 (10.7)	29 (7.4)	23 (10.8)
Missing	3 (0.5)	1 (0.3)	2 (1.0)	3 (0.8)	0 (0)
*Perceived comparative risk at baseline *^*α*^					
Less likely	180 (29.7)	129 (32.2)	51 (24.8)	116 (29.4)	64 (30.1)
About as likely	333 (54.9)	224 (55.9)	109 (52.9)	223 (56.6)	110 (51.6)
More likely	88 (14.5)	45 (11.2)	43 (20.9)	51 (12.9)	37 (17.4)
Missing	6 (1.0)	3 (0.8)	3 (1.5)	4 (1.0)	2 (0.9)
*Uncertainty about risk of breast cancer at baseline, mean (SD)*	1.7 (1.0)	1.7 (1.0)	1.7 (1.0)	1.7 (1.0)	1.7 (1.0)
Never	390 (64.3)	261 (65.1)	129 (62.6)	252 (64.0)	138 (64.8)
Rarely	66 (10.9)	43 (10.7)	23 (11.2)	41 (10.7)	24 (11.3)
Sometimes	96 (15.8)	60 (15.0)	36 (17.5)	63 (16.0)	33 (15.5)
Often	54 (8.9)	37 (9.2)	17 (8.3)	37 (9.4)	17 (8.0)
Missing	1 (0.2)	0 (0)	1 (0.5)	0 (0)	1 (0.5)
*Uncertainty about choices for breast cancer at baseline, mean (SD)*	1.59 (0.98)	1.6 (1.0)	1.5 (0.9)	1.6 (1.0)	1.6 (1.0)
Never	423 (69.7)	271 (67.6)	152 (73.8)	272 (69.0)	151 (70.9)
Rarely	58 (9.6)	38 (9.5)	20 (9.7)	38 (9.6)	20 (9.4)
Sometimes	77 (12.7)	58 (14.5)	19 (9.2)	52 (13.2)	25 (11.7)
Often	48 (7.9)	34 (8.5)	14 (6.8)	32 (8.1)	16 (7.5)
Missing	1 (0.2)	0 (0)	1 (0.5)	0 (0)	1 (0.5)

^¥^Significant at *p* < 0.05 comparing DBN versus no DBN

^α^Significant at *p* < 0.05 comparing BD awareness versus no BD awareness

“BD awareness”: women reported having heard of breast density, or having been told they had dense breasts. “No BD awareness”: women reported that they had not heard of breast density and had not been told they had dense breasts. “With DBN”: women with BI-RADS 3 or 4 on the baseline mammogram. “No DBN”: women with BI-RADS 1 or 2 on the baseline mammogram

As sensitivity analyses, to test the robustness of our findings to analysis specifications, we also estimated the average population association of DBN or awareness with psychological factors in the smaller sample of women that responded to each question across all waves using generalized estimating equations (GEE) models that included interaction for survey wave when appropriate. We also assessed the use of other proxies for notification status or awareness, including a four-level variable representing each combination of density and awareness, a variable representing the number of times a women has had mammographically dense breasts since 2013 prior to baseline, and the use of the dense status of the mammogram just prior to baseline. Finally, we assessed for interaction between DBN and BD awareness for each outcome and each wave using a cross product term and in stratified analyses. We used the same statistical approach to examine whether associations of DBN or awareness with psychological outcomes varied by educational attainment (high school or less vs. some college or more), health literacy (continuous), nativity (US-born vs. non-US-born), and preferred language of interview (English vs. Spanish). All analyses were conducted in SAS 9.4 (Cary, NC) using a significance level of *p* < 0.05.

## Results

### Descriptive analyses

We observed no statistically significant differences in sociodemographic and breast cancer-related characteristics between women who completed the baseline survey and those who completed each follow-up survey or all 3 surveys (data not shown) and therefore present the population characteristics of women who responded to the short-term survey in Table 2. Of the 607 women completing the short-term wave, 34% reported awareness of breast density, and 35% had a clinical report indicating that their baseline mammogram was classified as BI-RADS categories of heterogeneously or extremely dense and thus triggering DBN; these women were more likely to have BD awareness than women who had non-dense baseline mammograms and were therefore not eligible for DBN (57.8% vs. 21.1%). The mean age was 51.6 years, 63% were Spanish language dominant, 42% had completed a high school education or less, and about half had been called back after an earlier mammogram; 39% had at least one mammogram report with BI-RADS ascertained as dense composition since 2013, the year DBN was implemented in New York State, prior to the baseline mammogram report. This baseline report was used as our main DBN construct. Overall, most women (71%) only rarely or never worried about getting breast cancer and considered themselves to be at similar risk of breast cancer as compared to an average woman of their age (55%). Significant differences between either dense versus non-dense or aware versus unaware groups existed for all covariates presented in Table 1, except family history of breast cancer. Psychological outcomes reported at baseline were mostly consistent across BD awareness and DBN groups, with the exception of breast cancer worry, which was reported as “often” more frequently in women aware of BD, and perceived comparative risk of “less likely,” which was reported more frequently among women unaware of BD.

### Multivariable analyses

Adjusting for baseline psychological factors, only perceived absolute risk was associated with short-term awareness (Table 3). Further adjusted for DBN status, compared to women who were unaware of BD, women who were aware were more likely to report higher perceived risk at short-term follow-up. Specifically, aware women were marginally more likely to report high versus low risk (odds ratio (OR) 2.27, 95% confidence interval (CI) 0.99, 5.20), and significantly more likely to report moderate versus low risk (OR 2.19, 95% CI 1.34, 3.58) at short-term follow-up. Adjusted for BD awareness, women eligible for DBN (i.e., BI-RADS dense breasts classification) were less likely to report moderate versus low risk at short term (OR 0.62, 95% CI 0.40, 0.96). Neither association was significant at long-term follow-up after adjusting for outcome-specific covariates.

**Table 3:** Association of dense breast notification (DBN) and breast density (BD) awareness with psychological responses at short- and long-term follow-up

Psychological outcomes	Short-term follow-up		Long-term follow-up	
	With DBN versus No DBN **OR ( 95% CI)**	BD awareness versus No BD awareness **OR (95% CI)**	With DBN versus No DBN **OR (95% CI)**	BD awareness versus No BD awareness **OR (95% CI)**
*Breast cancer worry *^*α*^	*n* = 584		*n* = 589	
Model 1: High versus low	0.76 (0.52, 1.10)	0.80 (0.55, 1.17)	0.99 (0.69, 1.41)	0.88 (0.61, 1.28)
Model 2: High versus low	0.80 (0.54, 1.20)	0.87 (0.58, 1.31)	1.04 (0.70, 1.53)	0.87 (0.59, 1.30)
*Perceived absolute risk *^*β*^	*n* = 559		*n* = 566	
Model 1				
Moderate versus low risk	0.81 (0.54, 1.21)	1.78 (1.14, 2.78)	1.14 (0.76, 1.70)	1.27 (0.81, 2.01)
High versus low risk	1.21 (0.62, 2.37)	2.16 (0.99, 4.68)	0.71 (0.35, 1.42)	0.95 (0.43, 2.08)
Model 2				
Moderate versus low risk	0.62 (0.40, 0.96)	2.19 (1.34, 3.58)	1.07 (0.69, 1.63)	1.24 (0.76, 2.02)
High versus low risk	0.92 (0.45, 1.89)	2.27 (0.99, 5.20)	0.69 (0.33, 1.43)	1.08 (0.47, 2.47)
*Perceived comparative risk *^*β*^	*n* = 556		*n* = 583	
Model 1				
As likely versus less likely	1.08 (0.60, 1.94)	1.58 (0.99, 2.54)	1.29 (0.86, 1.94)	1.09 (0.69, 1.74)
More likely versus less likely	1.10 (0.71, 1.68)	0.90 (0.46, 1.74)	0.65 (0.35, 1.22)	0.66 (0.33, 1.32)
Model 2				
As likely versus less likely	0.94 (0.59, 1.49)	1.63 (0.43, 1.76)	1.30 (0.85, 2.0)	0.99 (0.60, 1.62)
More likely versus less likely	1.09 (0.59, 2.03)	0.87 (0.43, 1.76)	0.71 (0.36, 1.37)	0.73 (0.35, 1.52)
*Uncertainty about breast cancer risk*^*¥*^	*n* = 589		*n* = 612	
Model 1	1.11 (0.81, 1.53)	1.26 (0.91, 1.76)	0.91 (0.66, 1.26)	0.85 (0.60, 1.20)
Model 2	1.03 (0.73, 1.45)	1.25 (0.87, 1.78)	0.95 (0.68, 1.35)	0.87 (0.60, 1.25)
*Uncertainty about breast cancer screening choices*^*¥*^	*n* = 590		*n* = 606	
Model 1	1.13 (0.81, 1.57)	1.26 (0.87, 1.81)	1.02 (0.73, 1.42)	0.78 (0.53, 1.15)
Model 2	1.04 (0.73, 1.49)	1.24 (0.83, 1.83)	1.13 (0.78, 1.62)	0.74 (0.49, 1.12)

“BD awareness”: women reported having heard of breast density, or having been told they had dense breasts. “No BD awareness”: women reported that they had not heard of breast density and had not been told they had dense breasts

“With DBN”: women with BI-RADS 3 or 4 on the baseline mammogram. “No DBN”: women with BI-RADS 1 or 2 on the baseline mammogram

Model 1: Adjusted for outcome-specific covariates specified below and baseline outcome response

Model 2: Model 1 + mutual exposure (density for awareness model, awareness for density model)

Covariates selected based on association with outcome and either exposure:

Breast cancer worry: Gail 5-year absolute risk score, mammography callback, and history of breast biopsy

Perceived absolute risk: Gail 5-year absolute risk score, nativity status, health literacy, educational attainment, and history of breast biopsy

Perceived comparative risk: Gail 5-year absolute risk score, mammography callback, history of breast biopsy, and language of interview

Uncertainty, risk: Health literacy

Uncertainty, choices: Nativity status, history of breast biopsy, and health literacy

^¥^Cumulative Logistic regression

^α^Logistic Regression

^β^Multinomial Logistic Regression

Sensitivity analyses using the 4-level combination of DBN and awareness variables and stratification by DBN status yielded similar results. We also found similar results when we used the dense breast status of the last mammogram prior to the baseline mammogram, and when we used the number of dense breast mammograms since DBN implementation, instead of the baseline mammogram, as a proxy for notification status (data not shown). We observed similar findings when estimating the population average of the psychological factors in response to density or awareness using GEE models (OR 1.73, 95% CI 1.20, 2.50 for moderate vs. low absolute risk with awareness, Additional file 1: Table S1).

### Subgroup analyses

**Fig. 1:**
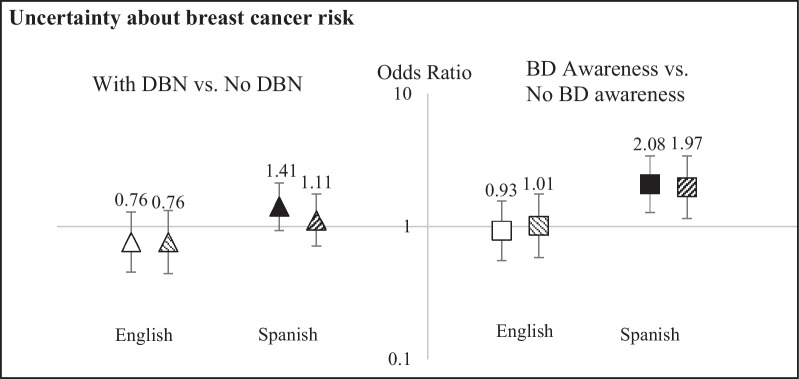
Effect modification by preferred language of interview on the associations of dense breast notification (DBN) (left panel) and breast density (BD) awareness (right panel) with uncertainty about breast cancer risk. At short-term follow-up. “BD awareness”: women reported having heard of breast density, or having been told they had dense breasts. “No BD awareness”: women reported that they had not heard of breast density and had not been told they had dense breasts. “With DBN”: women with BI-RADS 3 or 4 on the baseline mammogram. “No DBN”: women with BI-RADS 1 or 2 on the baseline mammogram. Solid marker: Model A: adjusted for outcome-specific covariates (health literacy) and baseline uncertainty about breast cancer risk, *p*_interaction, density_ = 0.06; *p*_interaction, awareness_ = 0.02. Hatched marker: Model A, plus mutual adjustment for other exposure, *p*_interaction, density_ = 0.07; *p*_interaction, awareness_ = 0.02

**Fig. 2:**
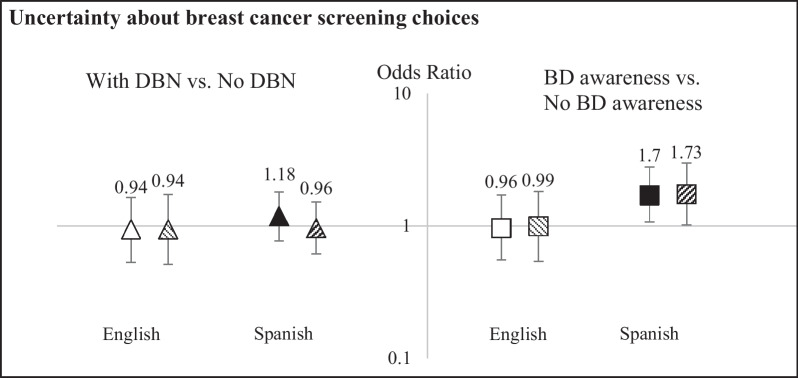
Effect modification by preferred language of interview on the associations of dense breast notification (DBN)  (left panel) and breast density (BD) awareness (right panel) with uncertainty about breast cancer screening choices. At short-term follow-up. “BD awareness”: women reported having heard of breast density, or having been told they had dense breasts. “No BD awareness”: women reported that they had not heard of breast density and had not been told they had dense breasts. “With DBN”: women with BI-RADS 3 or 4 on the baseline mammogram. “No DBN”: women with BI-RADS 1 or 2 on the baseline mammogram. Solid marker: Model A: adjusted for outcome-specific covariates (nativity status, history of breast biopsy, and health literacy) and baseline uncertainty about breast cancer screening choices, *p*_interaction, density_ = 0.4; *p*_interaction, awareness_ = 0.054. Hatched marker: Model A, plus mutual adjustment for other exposure, *p*_interaction, density_ = 0.4; *p*_interaction, awareness_ = 0.055

We observed no statistically significant multiplicative interaction between BD awareness and DBN, nor with either BD awareness or DBN and nativity or health literacy. However, both educational attainment and preferred language appeared to modify some of the associations for outcomes assessed at short-term follow-up (joint test *p* value < 0.05). We observed increased short-term uncertainty about breast cancer risk (OR 1.97, 95% CI 1.15, 3.39, Fig. 1) and uncertainty about breast cancer screening choices (OR 1.73, 95% CI 1.01, 2.97, Fig. 2) for women who were aware of breast density versus unaware specifically among women whose dominant language of interview was Spanish, but not for those whose dominant language was English (OR 1.01, 95% CI 0.58, 1.75 and OR 0.99, 95% CI 0.54, 1.81, respectively).

**Fig. 3:**
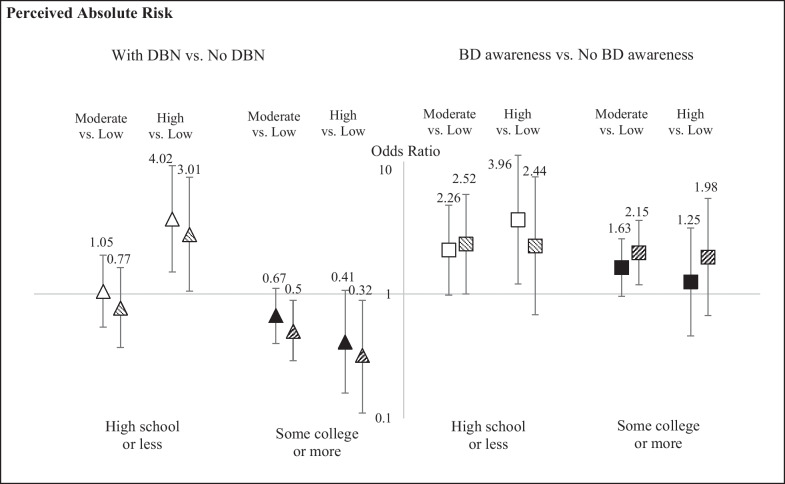
Effect modification by educational attainment on the associations of dense breast notification (DBN) (left panel) and breast density (BD) awareness (right panel) with perceived absolute risk. At short-term follow-up. “BD awareness”: women reported having heard of breast density, or having been told they had dense breasts. “No BD awareness”: women reported that they had not heard of breast density and had not been told they had dense breasts. “With DBN”: women with BI-RADS 3 or 4 on the baseline mammogram. “No DBN”: women with BI-RADS 1 or 2 on the baseline mammogram. Solid marker: Model A: adjusted for outcome-specific covariates (5-year absolute risk Gail score, nativity status, health literacy, educational attainment, and history of breast biopsy) and baseline perceived absolute risk, *p*_interaction, density_ = 0.01; *p*_interaction, awareness_ = 0.5. Hatched marker: Model A, plus mutual adjustment for other exposure, *p*_interaction, density_ = 0.01; *p*_interaction, awareness_ = 0.5

We also found an interaction between education and DBN, but not for BD awareness, for perceived absolute risk (Fig. 3). Specifically, women who had dense breasts relative to those who had non-dense breasts were significantly more likely to perceive their risk as high versus low (OR 3.01, 95% CI 1.05, 8.67) among women with a high school education or less, but this association was reversed among women with some college or higher education, who were significantly less likely to perceive their absolute risk as high (OR 0.32, 95% CI 0.11, 0.89) or moderate (OR 0.50, 95% CI 0.29, 0.89) versus low following DBN.

## Discussion

We investigated whether informing women of their dense breasts may increase women’s anxiety, perceptions of risk, worry, and uncertainty about breast cancer risk and screening, as suggested by qualitative studies [10, 11, 27]. In this sociodemographically diverse screening cohort, we found some evidence that awareness of breast density is associated with increased perceived absolute risk about 1–3 months following a screening mammogram, but that this increase is not observed over the longer term. This short-term association was independent of whether a woman had dense breasts on her own mammogram and hence had received notification. DBN was associated with decreased perceived absolute risk of breast cancer at short-term among women with some college education or greater, but increased perceived absolute risk among women with a high school or less education. We also observed effect modification of the associations of short-term uncertainty with breast cancer risk and screening choices by preferred interview language. Women who were aware of breast density and whose dominant language was Spanish reported greater uncertainty about both breast cancer risk and screening choices than those who were unaware, but no greater uncertainty was reported among those whose dominant language was English. There were also no statistically significant associations between awareness and dense breast status and any emotional or cognitive psychological responses at long-term follow-up (at approximately 1 year after baseline).

The particular outcomes for which we observed associations (absolute risk and uncertainty about breast cancer risk and screening choices) align with the content of the mandated DBN text in the New York State legislation [3, 5]. This text advises women that BD is a risk factor for breast cancer and that it poses limitations to detecting tumors on screening mammograms; these messages may, respectively, influence perceptions of personal risk of breast cancer and uncertainty about screening options shortly after receiving a mammography report containing DBN. Therefore, our results suggest that DBN may be effective in changing women’s cognitive responses that are specific to the information that is communicated to them through DBN. Uncertainty is inherent in most breast cancer screening and risk reducing interventions (e.g., genetic testing). The impact of this uncertainty on women’s psychological states and behavior may depend on personal characteristics such as race [12], but also tolerance to uncertainty around healthcare. While our main analysis suggested opposing directions of effect for DBN and BD awareness, this pattern of associations remained in our stratified analysis only among highly educated women. These effects are difficult to explain as we were not able to tease out more discrete combinations of DBN and BD awareness in our stratified analysis due to small sample sizes. It is possible that higher educated women who have received DBN may be more likely to be risk assessed and knowledgeable about their objective risk, and thus more likely to be reassured by a negative mammogram finding or DBN text; however, our findings and this explanation merit further investigation. Whether increasing perceived risk and uncertainty about breast cancer screening through DBN have adverse consequences for women’s psychological or behavioral outcomes remains unclear; improving the communication and clinical follow-up of the DBN information or clear screening guidelines for women with dense breasts may avoid negative consequences. Given our observed differences by women’s education and language, these efforts would also need to be tailored appropriately to reach all population subgroups and avert potential disparities in breast cancer screening and other outcomes. Additionally, though we did not have the power to explore this fully, women who were aware may have been more likely to remain stable in their reporting of their perceived absolute risk from baseline to short term versus decreasing during that time. This raises the possibility that the greater absolute risk we observed with awareness at short term may result from unaware women decreasing their reported perceived absolute risk after a negative mammogram. This should be explored in future studies.

The limited body of empirical literature to date has not established a large or consistent effect of notification on emotional or cognitive psychological outcomes although unlike this study, the majority of prior research was small qualitative studies immediately after reading DBN text, or examined cross-sectional associations, and focused on BD knowledge and awareness [6, 7, 13, 18, 28]. Cross-sectional studies may not be able to differentiate effects of DBN or awareness from baseline characteristics such as whether some woman have greater tendencies toward greater worry or risk perceptions. In our study, the use of repeated assessment of psychological outcomes starting with the mammography appointment and over the course of about 1 year allowed for adjustment of baseline psychological factors. We were also able to assess psychological outcomes at each timepoint and observed that the increased perceived risk at short term with BD awareness was not observed around the time of the next mammogram (i.e., 12–18 months following receiving mammography). Although cross-sectional studies show mixed results with DBN and perceived risk [6], several small studies assessing short-term responses to DBN have reported results in alignment with our findings. In one randomized control trial of women with dense breasts who either did or did not receive DBN, women who received DBN were less likely to report “a lot lower” perceived comparative risk (10.5% vs. 15.5% for women who did not receive DBN) both 4 weeks and 6 months after DBN receipt, while worry and absolute risk perception did not significantly differ with DBN [29]. Another study assessing women before and immediately after reading a DBN also found significantly greater perceived lifetime risk of breast cancer after notification [30]. Our finding that education and preferred interview language modified the associations adds new data for these outcomes and is broadly consistent with previous literature that found variation of the impact of DBN legislation on other factors such as awareness, knowledge, and understanding of breast density, including by educational attainment [6], suggesting that the messaging of the DBN text and breast density awareness in general may be complex or, not uniformly understood across subgroups, and that more tailored patient education may be appropriate. Interventions to accompany DBN such as additional written information or community health worker interpersonal interactions as a way to clarify DBN messaging, address women’s questions, and facilitate follow-up with health care providers are being evaluated and may be successful at increasing awareness of BD [15], but more research is necessary to assess the impact on psychological outcomes.

We ascertained DBN status using BI-RADS data for dense and non-dense breast classification levels retrieved from medical records, which provides an accurate measure of women’s eligibility for DBN. However, we did not assess whether women who had mammographically dense breasts at their baseline mammogram received, read, or absorbed the notification information. We also did not have a clear assessment of women who were newly made aware of breast density to see the impact of notification. Although we were able to use medical records to see how many times women had dense breasts on their mammogram in the past, this only accounted for mammograms done at the recruiting facility. Finally, a full exploration of multiplicative interaction with education and our 4-level combination density and awareness variable was impossible due to sparse cell size at this level. Our study was strengthened by the use of an ethnically and racially diverse cohort with a range of education levels, allowing us to explore effect modification by education and language. We were also strengthened by our use of validated constructs for psychological outcomes, and that we assessed these psychological outcomes in the same women at multiple timepoints.

## Conclusions

In a predominantly Hispanic screening cohort, awareness of breast density, regardless of one’s own dense breast status, appears to increase one’s perceived risk of breast cancer for a short time after undergoing mammography, but was otherwise not associated with emotional or cognitive psychological factors such as worry or long-term changes. Women with lower educational attainment had increased perceived absolute risk if they were eligible for dense breast notification, and women with a dominant language of Spanish showed increased short-term uncertainty about breast cancer risk and screening options if they had BD awareness. Taken together, these findings suggest that women with lower educational attainment or with language barriers or lower acculturation are at greater risk for short-term uncertainty among breast cancer choices with BD awareness and perhaps could specifically benefit from outreach clarifying the implications of breast density on screening choices.

## Supplementary Information

**Additional file 1.** **Table S1.** Average population association of dense breast notification (DBN) or breast density (BD) awareness with psychological factors.

## Data Availability

The data used in this current study are available from the corresponding author on reasonable request.

## Abbreviations

DBN: Dense breast notification
BD: Breast density
BI-RADS: Breast Imaging Reporting and Data System
OR: Odds ratio
CI: Confidence interval
