# Supplementary material for: Breast cancer worry, uncertainty, and perceived risk following breast density notification in a longitudinal mammography screening cohort
Source: Breast Cancer Res. 2022 Dec 21;24:95. doi: 10.1186/s13058-022-01584-2 (PMC9773500; doi:10.1186/s13058-022-01584-2)
Supplement: Supplementary file 1 — Additional file 1. Table S1. Average population association of dense breast notification (DBN) or breast density (BD) awareness with psychological factors. [file 13058_2022_1584_MOESM1_ESM.docx]

**Table S1.** Average population association of dense breast notification (DBN) or breast density (BD) awareness with psychological factors

| **Psychological Outcomes** | With DBN  Vs. No DBN  **OR ( 95% CI)** | BD awareness  Vs. No BD awareness  **OR ( 95% CI)** | |
| --- | --- | --- | --- |
| **Breast cancer worry** ^α^ (n=498) |  |  | |
| High vs. Low | 0.89 (0.67, 1.19) | 0.88 (0.64, 1.21) | |
| **Absolute Perceived risk** ^β^ (n=459) |  |  | |
| Moderate vs. Low risk | 0.88 (0.63, 1.23) | **1.73 (1.20, 2.50)** | |
| High vs. Low risk | 0.85 (0.48, 1.50) | 1.56 (0.84, 2.89) | |
| **Comparative Risk** ^β^ (n=469) |  |  | |
| As likely vs less likely | 1.16 (0.84, 1.59) | 1.21 (0.80, 1.81) | |
| More likely vs less likely | 0.89 (0.54, 1.46) | 0.78 (0.41, 1.49) | |
| **Uncertainty about breast cancer risk^¥^** (n=512) | | | |
|  | 1.03 (0.78, 1.35) | 0.91 (0.67, 1.23) | |
| **Uncertainty about breast cancer screening choices^¥^** (n=509) | | P_(interaction, visit*awareness)_ < 0.05 | |
|  | 1.09 (0.82, 1.45) | *Short-term:*  1.16 (0.79, 1.69) | *Long-term:*  0.74 (0.50, 1.09) |
| GEE model adjusted by baseline outcome response, outcome-specific covariates specified below, and mutual  exposure (density for awareness model, awareness for density model). For uncertainty about breast cancer screening choices, visit interaction significant for awareness (0.03), thus visit specific stratified estimates are presented. “BD awareness”: women reported having heard of breast density, or having been told they had dense breasts. “No BD awareness”: women reported that they had not heard of breast density and had not been told they had dense breasts. “With DBN”: women with BI-RADS 3 or 4 on the baseline mammogram. “No DBN”: women with BI-RADS 1 or 2 on the baseline mammogram.  Covariates and sample size:  Breast cancer worry: Gail 5-year absolute risk score, mammography callback, and history of breast biopsy.  Absolute perceived risk: Gail 5-year absolute risk score, nativity status, health literacy, educational attainment, and history of breast biopsy.  Comparative risk: 5-year absolute risk Gail score, mammography callback, history of breast biopsy, and language of interview.  Uncertainty risk: Health literacy  Uncertainty choices: covariates: Nativity status, history of breast biopsy, and health literacy.  **^¥^** Cumulative Logistic regression.  ^α^ Logistic Regression  ^β^ Multinomial Logistic Regression | | | |
